# Mock community as an in situ positive control for amplicon sequencing of microbiotas from the same ecosystem

**DOI:** 10.1038/s41598-023-30916-1

**Published:** 2023-03-11

**Authors:** Giulio Galla, Nadine Praeg, Filippo Colla, Theresa Rzehak, Paul Illmer, Julia Seeber, Heidi Christine Hauffe

**Affiliations:** 1 Conservation Genomics Research Unit, Research and Innovation Centre, Fondazione Edmund Mach, San Michele all’Adige, Italy; 2 Department of Microbiology, Universität Innsbruck, Innsbruck, Austria; 3 Institute for Alpine Environment, EURAC Research, Bozen, Italy; 4 Department of Ecology, Universität Innsbruck, Innsbruck, Austria

**Keywords:** Conservation biology, Microbial ecology, Environmental microbiology

## Abstract

Metataxonomy has become the standard for characterizing the diversity and composition of microbial communities associated with multicellular organisms and their environment. Currently available protocols for metataxonomy assume a uniform DNA extraction, amplification and sequencing efficiency for all sample types and taxa. It has been suggested that the addition of a mock community (MC) to biological samples before the DNA extraction step could aid identification of technical biases during processing and support direct comparisons of microbiota composition, but the impact of MC on diversity estimates of samples is unknown. Here, large and small aliquots of pulverized bovine fecal samples were extracted with no, low or high doses of MC, characterized using standard Illumina technology for metataxonomics, and analysed with custom bioinformatic pipelines. We demonstrated that sample diversity estimates were distorted only if MC dose was high compared to sample mass (i.e. when MC > 10% of sample reads). We also showed that MC was an informative in situ positive control, permitting an estimation of the sample 16S copy number, and detecting sample outliers. We tested this approach on a range of sample types from a terrestrial ecosystem, including rhizosphere soil, whole invertebrates, and wild vertebrate fecal samples, and discuss possible clinical applications.

## Introduction

The microbiota, or communities of bacteria, fungi, archaea, and viruses colonizing habitats in and on multicellular organisms or abiotic environments, is known to be fundamental for plant and animal health, as well as soil function^1–4^. Although our knowledge of the importance of microbiota is rapidly expanding^5–8^, including its role in human growth and development^9,10^, as well as in plant and non-human animal production^11,12^, metabolism^13^, and adaptation^14^, comparative studies of microbiotas from diverse organisms within the same ecosystem are still rare. Short read amplicon sequencing of the 16S rRNA gene allows microbiota composition and diversity to be characterized with unprecedented resolution^5,7^, and numerous protocols (http://www.earthmicrobiome.org/protocols-and-standards/16s/), technical guidelines^15^ and analytical pipelines are available for the metataxonomic analysis of a multitude of sample types (e.g. environmental:^16^; animal:^17^). However, the comparison of microbiota from multiple matrices (e.g. soil, whole invertebrates, vertebrate faeces) is not yet standardized, since available pipelines do not include controls for bias in DNA extraction, amplification and sequencing of microbial taxa in each sample and each sample type^15^. In addition, microbiotas can only be compared using the relative frequencies of identified microorganisms^18^, since taxon abundance cannot be estimated using conventional marker gene surveys. Two main solutions for providing positive controls of analytical bias have been suggested for monitoring experimental microbiota pipelines: the addition of a ‘mock community’ (commercial or custom populations of a known number of cells of a small number of well-characterized microbial taxa) into biological samples before DNA extraction (in situ MC); or the introduction of ‘PCR spike-ins’ of synthetic nucleic acids just before the amplification process (^19^; in situ SNA).

Up to now, MCs have mainly been used as controls to test the efficiency of new protocols^5,20–23^. However, as long as the organisms included in the MC are not components of the study microbiota, MC could be used as an in situ positive control by processing the sample and MC simultaneously, then computationally removing the MC sequences, allowing the reconstruction of the sample microbiota^24,25^. Similarly, SNA with negligible identity to known 16S rRNA gene sequences can be adopted as in situ positive controls^19,26^. An additional advantage of the MC is that the number (or abundance) of the 16S rRNA target gene copies can be estimated by normalizing the number of sample genes in relation to that of MC (although not the number of bacteria, due to variation in 16S gene copy number within and between prokaryotic species).

Importantly, despite the publication of several studies making use of such in situ positive controls for the quantification of microbial load and calibration of sequence reads^27–30^, there are currently no guidelines regarding suitable MC doses for samples other than bovine faeces, and the effects of MC on diversity estimates are completely unknown even for this sample type. Similarly, the effect of various doses of SNA on microbial diversity has not been studied.

**Figure 1:**
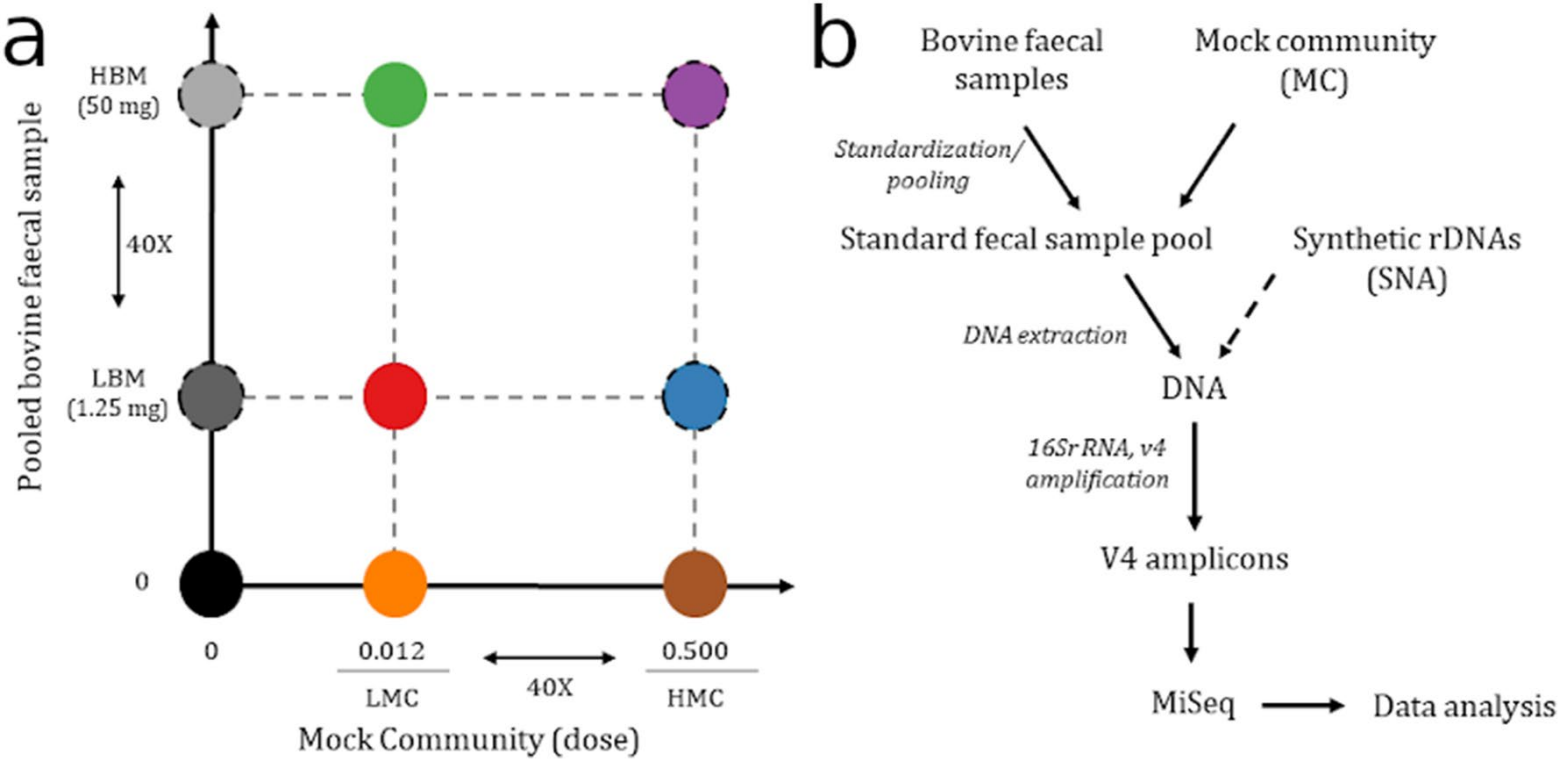
Experimental design. (**a**) combinations of mock community and pooled bovine fecal samples considered in the study. Each combination is marked with a different color. Colors reported in this figure match those used in the manuscript Figures. The black circle outline indicates the inclusion of synthetic 16S rDNA molecules (SNA) as PCR spike-ins. (**b**) schematic representation of the main methodological steps performed in this study. Briefly, bovine fecal samples were pooled into standardized fecal pools. Sample pools were supplemented with the mock community before DNA extraction. Synthetic rDNA molecules were added to DNA samples before PCR amplification. Libraries were sequenced on paired-end runs (2 × 250 bp), using an Illumina MiSeq sequencer. Data analyses included quality processing, generation of SVs and statistical analysis of sequencing data.

Here for the first time (to our knowledge), we used technical and biological replicates of ‘large’ and ‘small’ samples of pulverized bovine faeces (as proxies of field samples with high and low microbial biomass), and processed them with no, low or high doses of in situ MC or SNA to understand how in situ controls influence alpha and beta diversity indices of sample microbiota (Fig. 1). Throughout the manuscript, the microbial biomass of a sample is referred as to as ‘biomass’ and is defined as the total quantity of microorganisms amplifiable in a given sample weight. Based on these results, we tested the usefulness of MC for comparative microbial ecology, using biological samples from a variety of large and small organisms from a terrestrial ecosystem. We also verified that MC can be used to provide a direct measure of target gene number and abundance, with several ecological and clinical applications.

## Results

### Identification and quantification of MC sequence variants (MC-SVs)

The median number of raw sequence reads generated from bovine fecal pools (Pool identifiers: BP1, BP2 and BP3; Table 1) was 44,646, 54,817 and 24,693, respectively (Table S1), while the number of quality filtered sequence variants (SVs), ranged from 10,207 (Library ID: BP3_LBM_LMC_r7) to 58,075 (Library ID: BP2_HBM_LMC_r4). Linear mixed models using the Pool identifier as a random effect revealed no significant association between the percentage of quality filtered mapped reads (Table S1), MC dose (correlation between Pool (intercept) and MC dose: − 0.163) and sample biomass content (correlation between Pool (intercept) and biomass content: − 0.151), with 48.08% of total variance explained by the Pool identifier.

Unexpectedly, multiple (rather than single) MC-SVs matching the V4 region of *A. halotolerans* (4 SVs), *I. halotolerans* (3 SVs) and SNA (4 and 3 SVs for LC140931.1 and LC140933.1, respectively) were identified in all libraries including the spike-in controls. The same was true for the sequencing reactions made with the test samples (see below), which provided eight and three SVs matching the V3–V4 region of *A. halotolerans and I. halotolerans,* respectively*.* The alignment of V4 and V3–V4 MC-SVs to their reference sequences identified 29 and 19 polymorphic sites for *A. halotolerans* and *I. halotolerans*, respectively (data not shown). Inspection of the V4 fragments identified multiple clusters of complete MC-SVs (i.e. amplified with both primer pairs) sharing 98–100% identity with the publicly available reference sequences for these taxa (Fig. S1). Six out of 12 SVs matching *A. halotolerans* and four out of six SVs matching *I. halotolerans* were detected with an abundance about 100 times lower than the corresponding ‘primary’ MC-SVs (defined as those with the highest abundance and sharing 100% identity with the corresponding reference sequence; Fig. S1).

In the replicates of bovine fecal pools, the mean ratio of *I. halotolerans* (gram-negative) to *A. halotolerans* (gram-positive) SVs was 1.28 (± 0.22). This ratio was highly consistent for both in situ MC extracted in replicates of bovine fecal pools and MC-only controls (Table S1), and was significantly higher than 0.43, which is the expected value based on the number of cells included in the MC (manufacturer’s manual).

**Figure 2:**
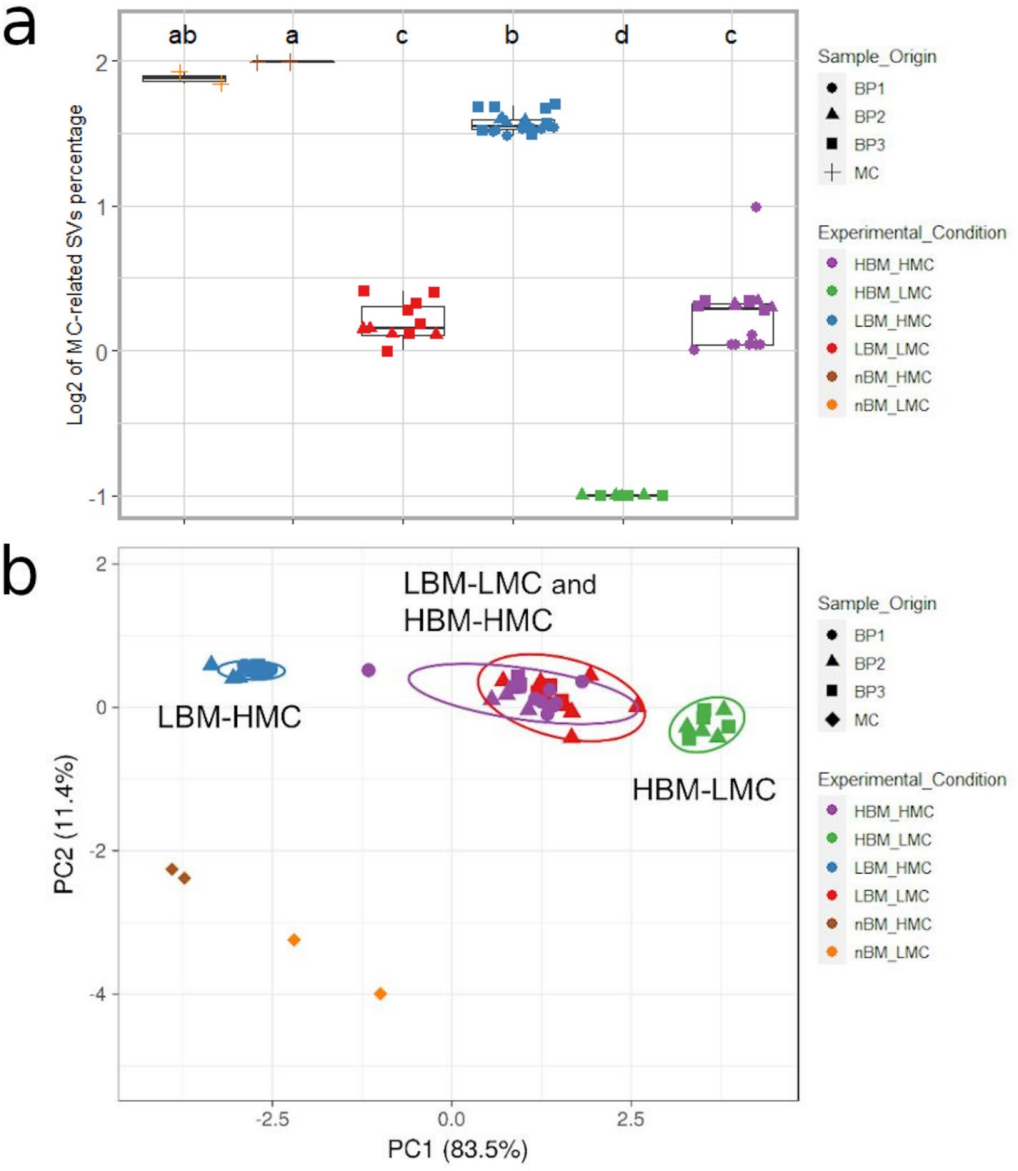
Clustering of samples based on the relative abundance of MC-SVs. (**a**) PCA of MC-SV sequence abundance. Original values were ln(x + 1)-transformed. Unit variance scaling is applied to rows; Singular value decomposition with imputation is used to calculate principal components. 95% prediction ellipses are shown for each combination of sample biomass and MC dose. (**b**) Proportion of MC-SVs compared to total SVs in each library. Results from Tukey HSD test on the ANOVA results indicated by (**a**–**d**).

The two MC doses (high and low) resulted in markedly different proportions of MC-SVs compared to the total number of reads in libraries generated from replicates of bovine fecal pools with high and low biomass content (Fig. 2a). The frequency of MC-SVs ranged from 0.1 to 49% in HBM-LMC (i.e. High Biomass and Low MC dose) and LBM-HMC (i.e. Low Biomass, High MC dose) libraries, respectively (Fig. 2a and Table S1). The PCA clustering of MC-SV abundances for each library (Fig. 2b) demonstrated a clear distinction between the abundance of MC in LBM-LMC (i.e. Low Biomass, Low MC dose) and HBM-HMC (i.e. High Biomass, High MC dose) libraries and those of LBM-HMC and HBM-LMC (Fig. 2b), as well as a clear separation between MC-only (control) libraries and libraries with in situ MC. Regarding in situ SNA (Table S1, Fig. S3) the highest abundances of synthetic DNAs were detected in LBM (i.e. Low Biomass) libraries (ranging from 0.1 to 3.5% quality filtered mapped sequences). In HBM (i.e. High Biomass) libraries, SNA abundances were lower and ranged from undetected to 0.04% (Table S1). The linear regression models used to correlate the log_2_ synthetic DNA copies to log_2_ SNA-SV counts generated R^2^ values ranging from R^2^ = 0.79 for the SNA molecule LC140931.1 (which was used in PCR amplifications with the highest concentration), to R^2^ = 0.37 for the SNA molecule LC140942.1 (used in PCR amplifications with the lowest concentration; Table S1 Fig. S3).

### Diversity estimates of bovine fecal replicates with and without in situ MC

**Figure 3:**
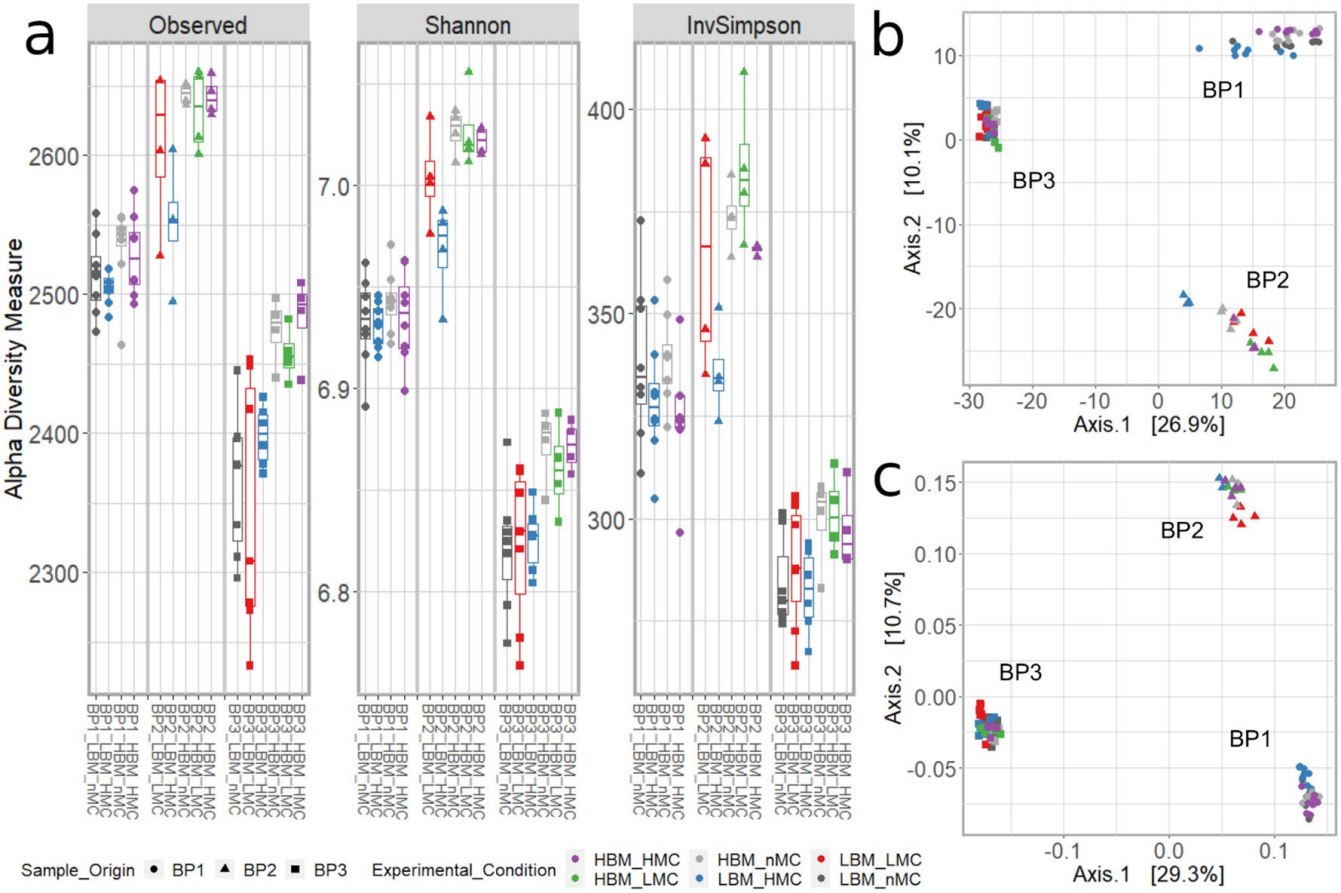
Diversity estimates for bovine fecal microbiota generated from sample pools BP1, BP2 and BP3 with high and low biomass and MC. (**a**) alpha diversity estimates. (**b**–**c**) Beta diversity estimates. PCoAs were generated by using Euclidean distances on CLR normalized datasets (**b**) and Bray–Curtis dissimilarity (**c**).

The incorporation of MC did not significantly affect richness (S), Shannon (H) or inverse Simpson (D_2_) alpha diversity estimates of replicates (Wilcoxon rank sum test *p-*values > 0.05, Table S2 Fig. 3a) compared to replicates with no added in situ MC. In addition, diversity differences between replicates of bovine fecal pools were consistent with their sample composition (Table S1), i.e., the pool generated from the highest number of samples (BP2) also had higher diversity estimates (Table 1).

**Table 1:** Summary of sample codes and characteristics. For each Pool (BP1, BP2, BP3 n = 3), the sample weight, mock community dose and number of technical replicates are reported.

Pool	Replicates	Weight (mg)^b^	MC dose^c^	Experimental conditions	Replicate code^d^
BP1	8	1.25	0	LBM-nMC	BP1-LBM-nMC
	8	1.25	0.5	LBM-HMC	BP1-LBM-HMC
	8	50.00	0	HBM-nMC	BP1-HBM-nMC
	8	50.00	0.5	HBM-HMC	BP1-HBM-HMC
BP2	4	1.25	0.012	LBM-LMC	BP2-LBM-LMC
	4	1.25	0.5	LBM-HMC	BP2-LBM-HMC
	4	50.00	0	HBM-nMC	BP2-HBM-nMC
	4	50.00	0.012	HBM-LMC	BP2-HBM-LMC
	4	50.00	0.5	HBM-HMC	BP2-HBM-HMC
BP3	7a	1.25	0	LBM-nMC	BP3-LBM-nMC
	7a	1.25	0.012	LBM-LMC	BP3-LBM-LMC
	7a	1.25	0.5	LBM-HMC	BP3-LBM-HMC
	4	50.00	0	HBM-nMC	BP3-HBM-nMC
	4	50.00	0.012	HBM-LMC	BP3-HBM-LMC
	4	50.00	0.5	HBM-HMC	BP3-HBM-HMC

^a^4 technical and 3 biological replicates.

^b^Low biomass (LBM) correspond to 1.25 mg of pooled bovine fecal sample; High Biomass (HBM) correspond to 50 mg of pooled bovine fecal sample.

^c^Fraction of the recommended dose. One dose (20 μl) includes 2 × 10^7^ cells of each of the two bacteria.

^d^Amplicon libraries were named by concatenating information for the pool (BP1, BP2, BP3), the sample biomass (LBM: 1.25 mg, HBM: 50.00 mg), the MC dose (nMC: no MC added, LMC: 0.0125 dose, HMC: 0.5 dose).

Principal coordinate analysis (PCoA) of replicates based on Euclidean distances and Bray–Curtis dissimilarities are shown in Fig. 3b,c, while PCoAs based on Unifrac distances are shown in Fig. S3. Permutational multivariate analysis of variance and PCoA based on Euclidean distance and Bray–Curtis dissimilarity metrics (Fig. 3, Table 2) as well as weighted and unweighted Unifrac distance (Fig. S2, Table S3) clustered libraries according to bovine fecal pool (Euclidean: R^2^ = 0.368, *p-*value = 0.001; Bray–Curtis: R^2^ = 0.405, *p-*value = 0.001; weighted Unifrac: R^2^: 0.702, *p-*value = 0.001; unweighted Unifrac: R^2^ = 0.221, *p-*value = 0.001). However, as shown in Fig. 3 and Fig. S2, and reported in Tables 2 and S3, variation in diversity/dissimilarity estimates across libraries with high and low MC and biomass (i.e. HBM-LMC, LBM-HMC, HBM-HMC and LBM, LMB) was also associated with the ratio between MC and sample biomass (Euclidean: R^2^ = 0.031, *p-*value = 0.013; Bray–Curtis: R^2^ = 0.022, *p-*value = 0.048; weighted and unweighted Unifrac: R^2^ = 0.41–0.019, *p-*value = 0.002–ns).

Again, across libraries generated from the same bovine fecal pool, the ratio between MC and sample biomass explained a significant fraction of variance in our estimates of Euclidean distance (R^2^ = 0.067–0.119, *p-*value = 0.017–0.001; Table 2), Bray–Curtis dissimilarity (R^2^ = 0.061–0.113, *p-*value ≤ 0.001; Table 2) and weighted UniFrac distance (R^2^ = 0.110–0.451, *p-*value = 0.046–0.001; Table S3. The incorporation of SNAs did not affect alpha diversity estimates of replicates (Fig. S3). Also, we found no variation in Bray–Curtis dissimilarity estimates associated with the presence or dose of SNA in PCR reactions (Fig. S3B; R^2^: 0.09599, *p-*value = 0.59).

**Table 2:** Permutational multivariate analysis of variance (PERMANOVA) of beta diversity estimates showing the influence of Pool identifier (Pool ID: BP1, BP2, BP3), and the ratio between MC and Biomass (MC-biomass ratio) in explaining overall variance in microbial communities. Statistical tests were carried out on the entire dataset (A, Pool ID: BP1-BP3) and on individual pools (B).

	Normalization strategy	Distance	Pool ID	Variable	F value	R^2^	Pr(> F)	Signif. Code
**A**	Centered log-ratio (CLR)	Euclidean	BP1-3	Pool ID	15.012	0.368	0.001	***
				MC-biomass ratio	2.527	0.031	0.013	*
				Residual		0.601		
	Rarefaction	Bray–Curtis	BP1-3	Pool ID	17.304	0.405	0.001	***
				MC-biomass ratio	1.902	0.022	0.048	*
				Residual		0.573		
**B**	Centered log-ratio (CLR)	Euclidean	BP1	MC-biomass ratio	1.892	0.119	0.001	***
				Residual		0.881		
			BP2	MC-biomass ratio	1.726	0.110	0.001	***
				Residual		0.890		
			BP3	MC-biomass ratio	1.359	0.067	0.017	*
				Residual		0.933		
	Rarefaction	Bray–Curtis	BP1	MC-biomass ratio	1.784	0.113	0.001	***
				Residual		0.887		
			BP2	MC-biomass ratio	1.346	0.088	0.001	***
				Residual		0.912		
			BP3	MC-biomass ratio	1.230	0.061	0.001	***
				Residual		0.939		

### 16S rRNA gene copy estimates and data transformation

**Figure 4:**
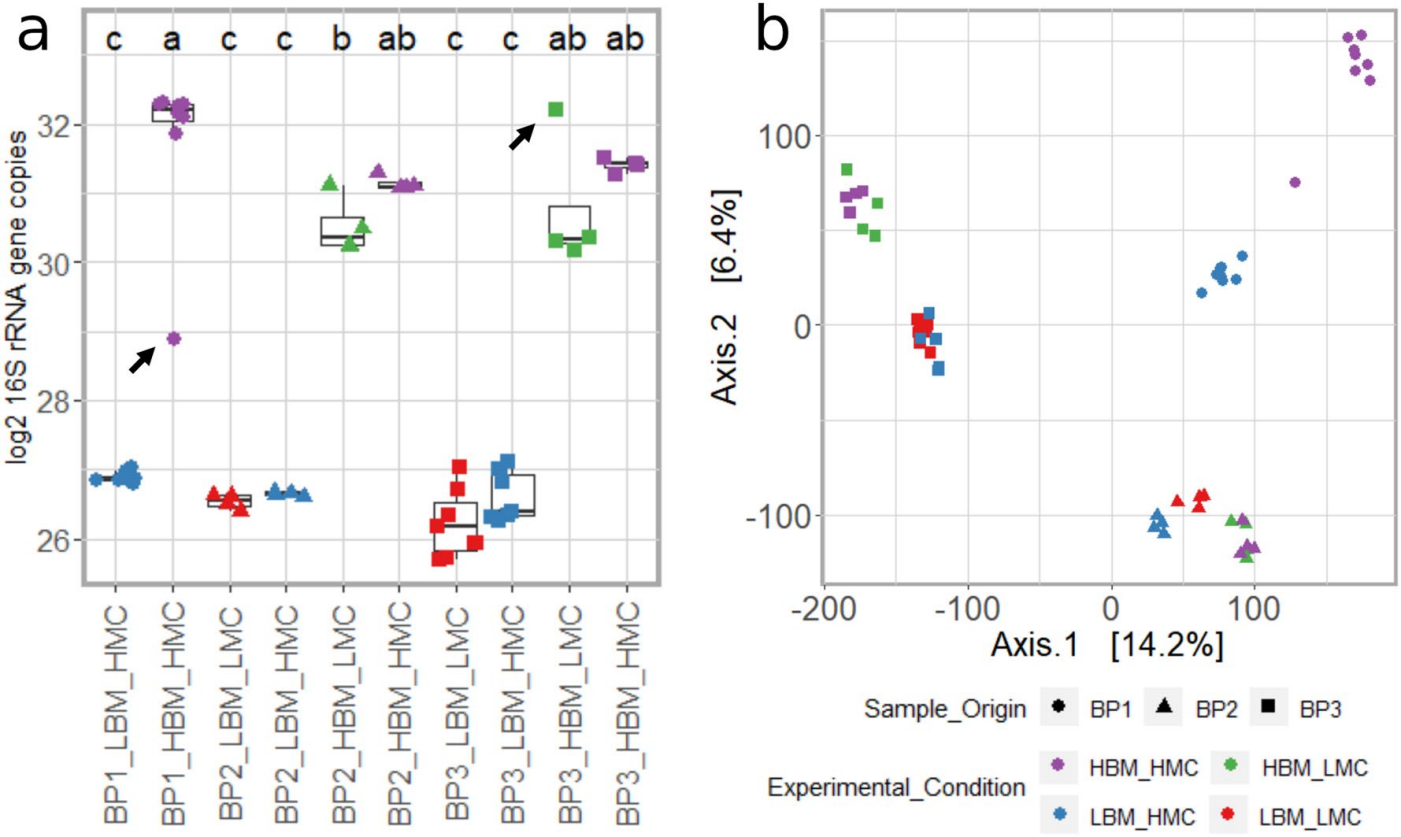
16S rRNA gene copy number and beta diversity estimates for bovine fecal microbiota with high and low biomass and mock community. (**a**) Log_2_ 16S rRNA gene copies estimated from the abundance of *I. halotolerans*—SVs in each library. the MC of the same library. Results from Tukey HSD test on the ANOVA results are indicated by (**a**–**c**). The black arrows indicate two potential outliers, characterized by an unexpected number of 16S rRNA gene copies: orange circle: 9.84 time fewer copies; purple square: 3.78 times more copies. (**b**) PCAs were generated by using Euclidean distance. SV counts were transformed according to the abundance of MC-SVs.

Log2 16S rRNA gene copies estimated from the abundance of *I. halotolerans* SVs (16S rDNA_j_) showed low variation between replicates of bovine fecal pools with the same experimental conditions (Table 1; Fig. 4a), although two libraries (BP1_HBM_HMC_r3 and BP3_HBM_LMC_r1) could be classified as outliers (black arrows in Fig. 4a). For each library processed with in situ MC, sample-SV abundances were transformed into MCnormSVij, by considering the estimated number of 16S rRNA gene copies in the corresponding library and the fraction of sample-SVs remaining after the removal of MC-SVs related to the gram-negative *I. halotolerans* (used as an indicator of the sample-microbial load). After this transformation, libraries clustered according to bovine fecal pool (R^2^: 0.191, *p-*value < 0.001; Fig. 4b, S4), as reported for untransformed datasets (Fig. 3, S2 and Table S3). However, in addition, transformed libraries also clustered according to their biomass, i.e. LBM and HBM libraries were represented by two separate clusters (R^2^: 0.045, *p-*value < 0.001, Fig. 4b). As observed for the untransformed data (Fig. 3b), the PCA in Fig. 4b indicated that microbial communities of bovine fecal pools BP1 and BP2 are more similar to each other than to BP3, which is consistent with their sample composition (Table S1).

### Diversity estimates and 16S rRNA copy number in rhizosphere soil, invertebrates, and mammalian fecal samples

MC-SVs were detected in all test samples, although their proportion compared to the total number of reads varied considerably (Table 3) across samples and MC doses. Despite the wide variety of sample types, at least one dose resulted in less than 2% MC-SVs being present in each library (Table 3); the only exception was Collembola libraries that had more than 35% MC-SVs.

**Figure 5:**
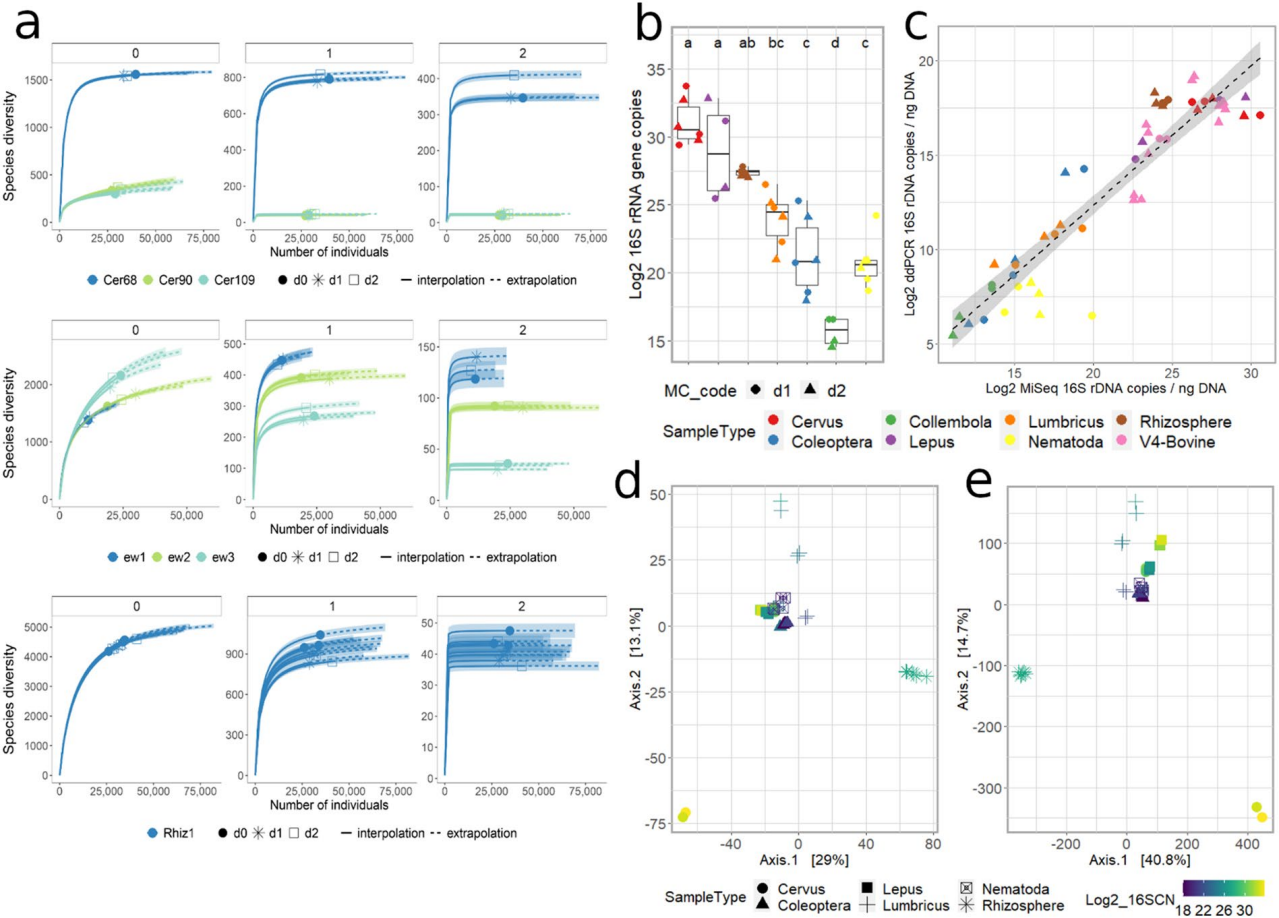
Diversity estimates and 16S rRNA copy number for test samples. (**a**) Sample-size-based rarefaction (solid lines) and extrapolation (dotted lines) sampling curves with 95% confidence intervals (shaded areas; based on a bootstrap method with 200 replications) separated by the diversity order [q]: q = 0 (species richness, left panel), q = 1 (Shannon diversity, middle panel) and q = 2 (Simpson diversity, right panel) for *C. elaphus* (upper plots), *Lumbricus* spp. (center plots), *Carex* spp. rhizosphere soil (bottom plots). MC doses are expressed as d0 (no MC added to the sample), d1: higher dose and d2: lower dose (please refer to Table 3 for additional details on MC doses for each sample type). (**b**) Log_2_ 16S rRNA gene copies estimated from the abundance of *I. halotolerans*-related SVs in the same library. (**c**) correlation between 16S rRNA gene copies estimated by ddPCR (y axis) and miSeq (x axis). The dotted line shows the corresponding linear regression line with 95% confidence interval (grey area). (**d**–**e**) Beta diversity estimates of test samples. PCAs were generated by using Euclidean distance on CLR normalized datasets. (**d**–**e)** plots were generated by using untransformed SV counts (**d**) and SV counts transformed according to the abundance of MC-related reads (**e**).

Diversity estimates for test samples are shown in Figs. 5 and S5. As reported for the bovine fecal pools, the main driver of diversity in mammalian fecal samples and large invertebrates at any MC dose was the individual, most clearly visible in *C. elaphus* (Fig. 5a), *L. europaeus* (Fig. S5), *Lumbricus* spp. (Fig. 5) and Coleoptera (Fig. S5). In addition, the R/E curves generated from libraries of the same sample type overlapped, regardless of MC dose (including no MC; Fig. 5; Table 3), in all test samples except Nematoda, for which we found high variability across pools and MC doses (Fig. S5). In addition, for test samples processed as technical replicates (*Carex* spp. rhizosphere soil and Collembola; Figs. 5a, S5), species richness and diversity were uniform and dose independent. Overall, the MC dose applied to the test samples did not affect Euclidean distances between their microbial communities, as indicated in Figs. S6 and 5d by the clear separation between sample types and low differentiation between replicates with various MC doses; again, only the small-sized animals Collembola and Nematoda showed significant variation in diversity across replicates with different MC doses (Fig. S6).

**Table 3:** Frequency of MC-SVs in each test sample.

Sample ID	Sample weight	[DNA] ± St.dev (ng/μl)	PCR template (ng/rxn)	MC dose: d1 (left), d2 (right) and generated MC reads (%)	
*Cervus elaphus*^c^				***0.1 ***^e^	*0.01*
Cer68	70 mg	80.3 ± 10.4 ng/μl	9	0.09%	0.01%
Cer90	40 mg	14.8 ± 3.6 ng/μl	9	0.79%	0.14%
Cer109	55 mg	13.2 ± 5.9 ng/μl	9	1.57%	0.07%
*Lepus europaeus*^c^				***0.15***	***0.015***
Lep638	50 mg	35.1 ± 6.8 ng/μl	9	0.67%	0.04%
Lep915	50 mg	-1.6 ± 0.4 ng/μl	0	28.95%	1.91%
*Lumbricus* spp.^c^				***0.001***	*0.0001*
ew1	10 mg	113.4 ± 22.6 ng/μl	100	2.94%	0.54%
ew2	20 mg	392.4 ± 29.4 ng/μl	100	0.12%	0.02%
ew3	25 mg	272.6 ± 24.4 ng/μl	100	0.55%	0.05%
Coleoptera^a, c^				***0.001***	***0.0001***
*Amara* spp.	7.5 mg	31.7 ± 3.2 ng/μl	60	0.41%	0.04%
*Cymindis* spp.	11 mg	113.1 ± 21.8 ng/μl	60	30.23%	5.48%
*Harpalus* spp.	14 mg	98.4 ± 24.6 ng/μl	60	6.86%	0.63%
Nematoda (bacterivorous)^b, c^				***0.0001***	*0.00001*
NemP1	30 ind	2.2 ± 0.3 ng/μl	13 ± 2	0.57%	0.08%
NemP2	28 ind	2.4 ± 0.7 ng/μl	15 ± 4	0.11%	0.03%
NemP3	31 ind	2.3 ± 0.8 ng/μl	14 ± 5	0.18%	0.01%
Rhizosphere soil (*Carex* spp.)^d^				*0.2*	***0.04***
Rhiz1	30 mg	7.4 ± 3.0 ng/μl	9	10.63%	2.66%
				10.65%	2.20%
				9.51%	2.88%
Collembola (entomobryomorpha)^d^				*0.001*	*0.0001*
Coll	~ 1 ind	3.8 ± 0.7 ng/μl	23 ± 4	84.89%	37.88%
				85.18%	37.29%

For each library (Sample ID), the table reports the corresponding taxonomic origin, the sample weight used for DNA extraction (Sample weight), the concentration and standard deviation of extracted DNAs ([DNA] ± St.dev), the MC dose and percentage of MC-SVs on the total number of reads (MC reads (%)). MC doses providing the best performances in terms of frequency of MC-SVs as well as alpha and beta diversity estimates are shown in bold.

^a^Processing of 0.5 *Harpalus* spp. (~ 25 mg) with 0.33 MC dose generated 0.94% ± 0.02 MC reads.

(data not shown).

^b^Processing of 7 individuals with 0.12 MC dose generated 0.98% ± 0.01 MC reads (data not shown).

^c^Biological replicates.

^d^Technical replicates.

e MC doses are in italics.

As shown in Fig. 5c, we found a strong linear correlation across libraries between 16S rRNA gene copies estimated from sequencing (using MC-SVs) and those measured with ddPCR (lm adjusted R^2^: 0.8545; *p-*value: 2.2e-16; Fig. 5c; Table S1). Variation in the number of gene copies across replicates extracted with different MC dose was only observed for Collembola (Fig. 5b,c). Given this finding (and previous findings above, i.e. high frequency of MC-SVs [Table 3], R/E curves [Fig. S5]), this taxon is not shown in Fig. 5e. In agreement with the results for bovine fecal pools, PCAs generated from untransformed (Fig. 5d) and transformed (Fig. 5e) SV counts of test samples had similar clustering patterns, except for *L. europaeus*, where samples with low and high biomass, as estimated from the frequency of MC-SVs and 16S rRNA gene copy number, clustered together for the untransformed, but not for transformed SV counts (compare Fig. 5d,e).

## Discussion

To the best of our knowledge, this is the first study reporting the effect of an in situ positive control (a mock bacterial community added to a sample before DNA extraction) on microbiota diversity estimates for a range of biological samples from the same ecosystem. Our results demonstrated that an appropriate MC added directly to a sample before extraction can function as an effective positive control with negligible effect on alpha and beta diversity estimates; moreover, the in situ MC allowed us to estimate the sample 16S rRNA gene copy number, with a number of potential applications. Using biological and technical replicates of bovine faecal samples, we examined the possible application of this commercial MC as an in situ positive control for amplicon sequencing of microbiotas without affecting sample diversity and composition indices. Using our results, we then tested a suitable range of MC doses on a wider collection of environmental and invertebrate samples.

The Synthetic Nucleic Acids (SNA) that we tested on a subset of bovine fecal pools were designed in such a way to be distinguishable from the sequences of known bacteria populating animal and environmental microbial communities^31^. Conversely, the choice of an MC suitable as in situ positive control relies on the lack of overlap between taxa included in MC and sample microbial communities. Despite the lack of knowledge regarding the composition of many environmental microbial communities, the isolation of *I. halotolerans* and *A. halotolerans* from environments characterized by high salinity^32,33^, suggests that the MC used here could also be applied as a positive in situ control to a wide range of terrestrial and host-associated contexts (e.g. sample types) where these taxa are unlikely to be found. Our study confirmed that the two taxa included in this MC were not identifiable within the microbiota of bovine fecal pools used here, nor were detected in rhizosphere soil (present study and^34^), fecal pellets of *C. elaphus* and *L. europaeus*, macrofauna (*Lumbricus* spp., Coleoptera), mesofauna (Collembola) or microfauna (Nematoda) samples. Moreover, *I. halotolerans* and *A. halotolerans* were not identified in the dataset from a previous study on bovine fecal microbiota^35^, nor were they detected in fecal samples of polar bears (*Ursus arctos*) inhabiting Arctic coastal regions and marine environments^36^.

Our analyses identified multiple SVs matching *I. halotolerans* and *A. halotolerans* with highly consistent frequencies across technical and biological replicates of all sample types tested here (Fig. S1). If this inflation in SV number were due to the presence of sequencing errors, it could have been a serious issue since this implies that sample microbiota diversity estimates would also have been artificially inflated. However, this is unlikely since (1) alpha diversity estimates in our bovine fecal samples were in line with that reported in previous studies using similar denoising strategies^35,37–39^; (2) the alignment of the V4 regions of the MC-SVs from both the bovine pools (amplified for V4 only) and test samples (amplified for V3–V4) generated separately identified the same SVs (Fig. S1), suggesting that they can be imputed, at least in part, to biological sequence variation present in the cultured bacteria included in the MC. Multiple MC-SVs implies the coexistence of non-identical 16S rRNA gene copies in the bacterial genomes and/or genetic variability among strains of the two reference species (i.e. inter-genomic variation)^40,41^. We could not confirm which scenario was correct as only a single 16S rRNA gene sequence for each of the two taxa is currently available in public databases, with no reports of inter- and intragenomic variation for these taxa, either in the literature or by the manufacturer of the MC.

Moreover, some variation in MC-SVs might be due to low frequency sequencing errors, for example, the MC-SVs matching *A. halotolerans* and *I. halotolerans* with an abundance about 100 times lower than the corresponding primary MC-SVs (Fig. S1) were well below the expectation of a single divergent 16S rRNA gene copy per bacterial genome^40^ (1/7 for *A. halotolerans* and 1/3 for *I. halotolerans*) suggesting sequencing errors if the MC derived from a single cellular strain. However, sequence diversity of 16S rRNA genes within individual prokaryotic genomes has been estimated to range from 0.06 to 20.38%, and such a wide range complicates the definition of a minimum identity threshold useful to distinguish true variants from sequencing errors, particularly for short reads amplicons. Although we cannot rule out the possibility that multiple strains with different abundances are included in the commercial MC adopted in this study, very low abundances are probably consistent with low frequency errors^42^ generated during PCR amplification and library preparation, and escaping the denoising data analysis ^43^. Further studies are needed to clarify intra- and inter-genomic variation in these taxa, to resolve this issue.

Since SNAs are only added to PCR reactions, they cannot be used to estimate DNA extraction efficiency. Instead, MC proved a particularly useful indicator of DNA extraction bias highlighting that the ratio between the two MC taxa was greater than expected across libraries of both bovine fecal pools (processed with the QIAamp® Fast DNA Stool Mini kit (QIAGEN)) and test samples (processed with the NucleoSpin® Soil mini kit (Macherey–Nagel)) confirming a well-documented issue in metataxonomic studies: a low extraction efficiency of gram-positive bacteria (in this case, *A. halotolerans*;^17^). As DNA extraction has been shown to be the main contributor to the distortion of bacterial abundance from their original values by altering, for instance, the abundance of taxa more difficult to lyse^17,44^, particular care should be played in the selection of the most appropriate DNA extraction method for metataxonomic studies. In the case of environmental or non-invasive fecal samples, we recommend the adoption of DNA extraction methods which allow managing the PCR inhibitors frequently associated with these sample type, while at the same time allowing the efficient breakdown of gram-positive bacteria cell walls with methods such as bead beating^17^ or enzymatic lysis (e.g. lysozyme).

By comparing libraries processed with or without MC, we showed that adding MC directly to samples before extraction did not affect sequencing performance (Table S1), or alpha diversity indices (Fig. 3a), even in test conditions in which MC-SVs were among the most abundant. Among alpha diversity estimates, richness estimates were also unaffected, indicating a neglectable effect on rare taxa, whose variation is typically emphasized by this diversity index. However, the clustering of LBM-HMC bovine fecal pools in the PCAs/PCoAs (Fig. S2) and PERMANOVA analyses (Tables 2, S3) suggested that a relative abundance of MC-SVs higher than 30% has the potential to influence beta diversity estimates. This effect may have been due to ‘competition’ between taxa during amplification and sequencing reactions, leading to high variability in the abundance of rare taxa^45^. This conclusion was corroborated in test samples such as Collembola where MC-SVs with replicates of the same pool ranged from 37 to 85% (Tables 3, S4). Instead, where the MC-SVs did not exceed 10% no changes were observed in sample diversity of bovine fecal pools (Fig. 5), or test samples (fecal pellets of red deer, whole beetles and earthworms, rhizosphere soil). Based on our results, we recommend using an MC dose so that MC-SVs are 1–10% filtered sample-SVs. The MC doses reported in Table 3 can be used as a reference and considered as starting points for future studies. While estimating the sample weight of biological samples is fairly straightforward, especially for soil, rhizosphere and fecal samples, researchers are encouraged to make preliminary calibration experiments with serial dilutions of in situ positive controls to find the dose suitable for their sample type and experimental design, especially if the freshness of samples cannot be guaranteed (as was the case for the *L. europaeus* samples), or if working with a wide variety of invertebrates.

Following the adoption of the V4 region as target marker in a number of international projects focusing on human, environmental and host-associated microbiota^9,16,46–49^ and the publication of a library preparation protocol based on the V3–V4 region by Illumina^50^, these hypervariable regions became very popular and widely adopted in metataxonomy studies. However, primer combinations targeting other hypervariable regions of the 16S rRNA gene^40^ are available^40^ and widely adopted. Of note, switching to a different hypervariable region might result in the lack of amplification of certain taxa ^40,51,52^, e.g. as reported for a V1–V2 primer pair which failed the detection several Bifidobacteriales^53^, eventually resulting in changes in the observed competition between host and MC microbial communities for PCR and sequencing resources. Therefore, although the two hypervariable regions tested in this study (V4 in bovine pools and V3–V4 in all test samples) displayed comparable performances across 16S gene copy number estimates made with ddPCR (MC independent) and MiSeq data (MC dependent), it is possible that choosing another hypervariable region might result in a different effect of MC on the total number of sequenced reads, particularly if the alternative primer set is associated with a lack or excess of amplification for a significant fraction of sample DNAs compared to the V3–V4 and V4 regions.

After we confirmed that the extraction efficiency of the gram-positive MC taxon *A. halotolerans* was biased, we used the gram-negative *I. halotolerans* as the reference taxon to estimate the 16S rRNA gene copy number in each sample. However, it should be noted that other studies using the same MC as an in situ positive control have adopted *A. halotolerans* as the reference taxa^28^. We also showed here that the strong correlation between gene copy number estimated by the sequencing data and those derived from ddPCR assays (Fig. 5), suggests that MC is an efficient alternative to qPCR, ddPCR^54,55^ or flow cytometry^56^ for estimating overall microbiota abundance, which avoids analysing samples twice, and would be particularly useful in the case of rare, unique or medically important samples with very small biomasses. In addition, the number of 16S rRNA gene copies together with beta diversity estimates of transformed SVs facilitated the identification of samples that were outliers in terms of biomass content, MC dose and/or DNA extraction efficiency (Fig. 4). In fact, while several normalisation strategies are available (e.g. rarefaction and CLR^57,58^) for tuning library size and taxa abundances between different samples to facilitate their comparison, these methods do not relate sample biomass to microbial load. Instead, our approach showed that, when the proportion of *I. halotolerans* SVs was used to transform sample-SV abundances, PCoAs of the transformed data exposed the impact of sample biomass and microbial load on beta diversity estimates (Figs. 4 and 5;  5d,e).

We believe that the use of MC as an in situ control will prove useful in the study of microbial ecology, but also in clinical studies. For example, clinical samples such as buccal^59^ and skin swabs^60^ have microbial biomasses of the same order of magnitude as small invertebrates like Nematoda or Collembola; hence, using in situ MC to calculate gene copy number in clinical samples would facilitate detection of dysbiosis, which depends on both community composition and absolute number of microrganisms, as seen in several human^56,61^ and plant^62^ diseases.

## Materials and methods

### In situ positive controls: mock community and synthetic DNA molecules

The ZymoBIOMICS™ Spike-in Control I (Cat No. 6320; EuroClone, Irvine, CA, USA) was chosen as the mock community (MC) for our study. We considered it the most suitable as it is composed of *Imtechella halotolerans* and *Allobacillus halotolerans*: ACC: NR116607.1, NR117181.2), which were isolated from marine habitats and, therefore, were unlikely to be present in our samples from terrestrial ecosystems. A single MC dose (20 μl, defined by the manufacturers) includes 2 × 10^7^ cells, corresponding to 6.0 × 10^7^ (*I. halotolerans*) and 1.4 × 10^8^ (*A. halotolerans*) 16S rRNA gene copies (ratio between *I. halotolerans* and *A. halotolerans* 16S rRNA gene copies: 0.43)*.* In addition, four SNA sequences corresponding to the 16S DNA V4 region were adopted as PCR spike-ins (accession number: LC140931.1, LC140933.1, LC140939.1, LC140942.1; GenScript Biotech (Netherlands;^19^). The target region was amplified using the two primers M13F (GTAAAACGACGGCCAG) and M13R (CAGGAAACAGCTATGAC), purified with the QIAquick PCR Purification Kit (QIAGEN) following manufacturer’s instructions, verified with Sanger sequencing, and quantified with the kit Quant-iT ™ dsDNA High-Sensitivity Assay (Thermo Fisher Scientific) using a Spark® multimode microplate reader (Tecan, Switzerland). For each amplicon, the theoretical number of molecules included in the PCR spike-in was inferred from the estimated DNA concentration and by considering the molecular weight of each SNA. Based on these estimates, the SNA mixture sp10st was composed of LC140931.1: 375,000 DNA fragments/μl; LC140933.1: 75,000 DNA fragments/μl; LC140939.1: 15,000 DNA fragments/μl; LC140942.1: 3000 DNA fragments/μl. SNA mixtures sp1st and sp0.1st were 1:10 and 1:100 dilutions of sp10st.

### Sample preparation, standardization and DNA extraction

Bovine fecal samples were collected from eight Pezzata Rossa Italiana heifers pastured on two sites at 2000 m a.s.l. (Vinschgau Valley, Province of Bolzano, Italy; site code LTER_EU_IT_097 ‘Val Mazia/Matschertal’). Freshly deposited cow pats were sampled using sterile tweezers; approximately 50 g of fecal matter were collected from three points per pat, placed in sterile 50 ml polypropylene tubes and stored on dry ice for up to 8 h before being transferred to the Fondazione E. Mach (Trento, Italy) where they were stored at -80 °C until pooling and DNA extraction. To make technical replicates, bovine fecal samples were combined into three ‘pools’ (BP1, BP2, BP3; Table S1) as follows: for each pool, approximately 0.5 g of each frozen fecal sample were placed together in a sterile mortar containing liquid nitrogen and ground to powder with a sterile pestle. Approximately 200 mg of this powder were mixed with 4 ml of preheated InhibitEX Buffer from the QIAamp® Fast DNA Stool Mini kit (QIAGEN Inc., Valencia, CA, USA), vortexed and split into three 1 ml subsamples (hereafter, ‘high biomass’, HBM) and three 25 μl subsamples (‘low biomass’, LBM) (Tables 1 and S1). Since the LBM subsamples were generated using 1/40th of the fecal material used for HBM subsamples and both were generated from the same powder, the difference in measured sample weight was used as a proxy for the difference in subsample microbial biomass.

The MC was added to each subsample in one of two doses: half a dose (10 μl, hereafter high mock community, HMC) or 1/40th of this (0.25 μl, hereafter low mock community, LMC) (Fig. 1a, Table 1). DNA extraction followed the manufacturer’s protocol for the isolation of DNA from stool for pathogen detection. A minimum number of four technical replicates were generated by processing 200 μl aliquots of the lysate supernatant independently from step 6 of the kit protocol. Negative controls to detect contamination during DNA extraction (lysis buffer only: no fecal material and no MC) and PCR amplification (PCR buffer only: no DNA template); positive controls for MC DNA processing (MC only: no fecal sample); and positive controls for fecal DNA processing (fecal sample only: no MC) were added to the analyses from the extraction step, amplified and sequenced. A summary of this experimental design can be found in Fig. 1a,b and Table 1.

### Test samples

In order to verify our comparative microbiota approach on a wide array of sample types containing microbiota from large and small organisms from a terrestrial ecosystem were collected from the same site as the bovine samples described above: rhizosphere soil from *Carex* spp. (N = 9 samples, all technical replicates of a single soil sample); whole ground beetles (Carabidae spp.; N = 9, three whole individuals with three technical replicates each); earthworms (*Lumbricus* spp.; N = 9, three individuals with three technical replicates each); springtails (CollembolaN = 6, a single pool of six whole individuals divided into six technical replicates); roundworms (Nematoda spp.; N = 9 pools of 30 whole individuals each); fecal pellets of red deer (*Cervus elaphus*; N = 9, three fecal pellets with three technical replicates each) and fecal pellets of European brown hare (*Lepus europaeus*; N = 6, two fecal pellets with three technical replicates each). Details of sampling methods, sample mass, pool composition and MC dose, as well as DNA extraction and amplification strategies are reported in Table S4 and supplementary methods.

### 16S rRNA gene amplification, library preparation and amplicon sequencing

The amplification of bovine fecal DNA was performed as described in (https://earthmicrobiome.org/protocols-and-standards/16s/), by using the FastStart High Fidelity Enzyme Blend (Roche Applied Science), with the two primers 515F_ILL^63^ and 806R_ILL^64^. High-throughput sequencing of the amplicon libraries using Illumina technology were performed at the Genomics Platform, Fondazione E. Mach. The 94 amplicon libraries were sequenced on three Illumina MiSeq Standard Flow Cells (Illumina, UK) using 500 cycle V2 reagents and with a minimum depth of 30,000 reads per sample.

### Data analysis

Bioinformatic pre-processing of all fastq files was carried out using MICCA^65^. Sequences were filtered by considering an expected error of 0.75 and a minimum sequence length of 200 bp. The generation of sequence variants (SVs) and SV counts were performed with UNOISE3^66^ implemented in MICCA, and subsequent statistical analyses were performed with R^67^. The sample BP3_LBM_HMC_r4 was removed from the dataset due to low sequencing performance. SVs matching the MC 16S rRNA gene sequences and the synthetic DNAs (SNA) were confirmed with BLAST (https://blast.ncbi.nlm.nih.gov/Blast.cgi), noted and removed from all relevant datasets before performing subsequent steps. Multiple sequence alignments of *A. halotolerans* and *I. halotolerans* MC-related SVs amplified using V4 and V3–V4 primer pairs with the publicly available sequences of *A. halotolerans* (NR_116607.1) and *I. halotolerans* (NR_117181.2) were performed with the software MUSCLE (https://www.ebi.ac.uk/Tools/msa/muscle/). Multiple sequence alignments were then imported in Geneious Prime (Dotmatics) and trimmed to the V4 region.

The association between MC dose and number of quality filtered reads was tested using the R package lme4^68^ with the following formula lmer(mapped_reads ~ MC_dose + Biomass_Content + (1|Pool_ID), data = data).

The percentage of MC-SVs in each bovine fecal library was compared across pools with a one-way ANOVA and Tukey's test with the agricolae R package^69^. To generate the Principal Component Analysis (PCA) plots based on the abundance of MC-SVs (Fig. 2b), SV counts were normalized according to^70^. The PCA plot based on the abundance of MC-SVs was generated by using the web tool ClustVis^71^. The correlation between the number of synthetic DNA copies in the PCR template (reported as Log2 transformed, y axis) and the observed number of SNA-related SVs (Log2 x + 0.1 transformed, x axis) was tested using a linear regression model in Windows Excel. To compare the diversity indices of libraries with different sequencing depths, we employed the centered log-ratio (CLR) normalization strategy. Before converting the SVs counts to CLRs using the ‘codaSeq.clr’ function of the R package CoDaSeq^72^, we added an offset of 1 to the whole count matrix. Using the R package phyloseq^73^, CLR values were used to calculate Euclidean distances and the ordination of samples, otherwise counts were rarefied to 99% of the minimum sample depth in the dataset (10,093 reads per sample). Standard alpha and beta diversities were estimated with the R package phyloseq^73^. Significant differences in alpha diversity estimates across groups of samples were tested with Wilcoxon rank sum tests^73^. Permutational ANOVA (PERMANOVA) statistical tests were performed with Pool ID and MC:biomass ratio as independent variables using the function ‘adonis2’ with 999 permutations in the R package vegan^74^. The MC:biomass ratio was defined as either 1 (as in HBM-HMC and LBM-LMC), 0.025 (for LBM-HMC) and 40 (for HBM-LMC). . Plots were generated with the R package ggplot2^75^.

In order to use MC-SVs to estimate the total number of 16S rRNA gene copies (GCN) in the jth library, 16S rDNA_j_ was estimated as: N_total_reads _j_/SV_I.halotolerans _j_ * MC_dose _j_, where N_total_reads _j_ is the total number of quality filtered reads for the jth library, SV_*I. halotolerans*_j_ is the abundance of SVs related to *I. halotolerans* in the jth library; and MC dose_j_ is the dose of mock community used in library j (ZymoBIOMICS™ Spike-in Control I manual). Log_2_ 16S rRNA gene copies estimated from the abundance of *I. halotolerans* SVs in each library were compared across libraries using one-way ANOVA and Tukey's Test with the R package agricolae^69^.

The transformation of sequence counts for each SV or MCnormSV_ij_ (i) in library (j) according to the total number of 16S rRNA gene copies and biomass content was calculated as follows: (SV_i j_/counts_j_) *16S rDNA_j_ *(1-(SV_*I. halotolerans* j_/counts_j_)), where MCnormSV_ij_ is the normalized abundance of the ith SV in the jth library, SV_ij_ is the abundance of the ith SV in the jth library, counts_j_ is the number of sequences in the OTUtable for jth library, 16S rDNA_j_ is the total number of 16S rRNA gene copies in the jth library and SV_I. halotolerans j_ is the abundance of SVs related to *I. halotolerans* in the jth library.

## Supplementary Information

Supplementary Information 1.

Supplementary Information 2.

Supplementary Information 3.

## Data Availability

The raw sequence data is deposited in the NCBI Sequence Read Archive (SRA) under the BioProject IDs PRJNA703791 (https://www.ncbi.nlm.nih.gov/sra/PRJNA703791) and PRJNA734187 (https://www.ncbi.nlm.nih.gov/sra/PRJNA734187).
