# Supplementary material for: Mock community as an in situ positive control for amplicon sequencing of microbiotas from the same ecosystem
Source: Sci Rep. 2023 Mar 11;13:4056. doi: 10.1038/s41598-023-30916-1 (PMC10008532; doi:10.1038/s41598-023-30916-1)
Supplement: Supplementary file 1 — Supplementary Information 1. [file 41598_2023_30916_MOESM1_ESM.docx]

**Sampling, DNA extraction and amplification of the V3-V4 region of the 16S rRNA gene for test samples**

Faecal pellets of European brown hare (*Lepus* spp.) and red deer (*Cervus elaphus*) along with *Carex* spp. rhizosphere soil and invertebrate taxa (*Lumbricus* spp., Coleoptera, Collembola, Nematoda) considered in the test samples were collected in Val Mazia/Matschertal, South Tyrol, Italy (site code LTER_EU_IT_097, 46.6928°, 10.6157°) between 15/07/2019 and 15/08/2019.

Fresh faecal pellets of European brown hare (*Lepus* spp.) and red deer (*Cervus elaphus*) were collected using sterile tweezers in sterile 15 ml polypropylene tubes and stored on dry ice for up to 8 hours before being transferred to the Fondazione E. Mach (Trento, Italy) and stored at -80°C until pooling and DNA extraction. Faecal pellets were dissected in a biological cabinet with sterile forceps and blades. The samples were generated by dissecting the central region of the pellet along the longitudinal axis (avoiding the two terminal regions) and were made in such a way as to sample both the outer and the inner region of the pellet. For both European brown hare (two pellets) and red deer (three pellets), three technical replicates were generated by performing multiple dissections from the same pellet.

Sampling of *Carex* spp. with entire root system and rhizosphere soil collection have been performed according to ^[1],[2]^. A single soil sample was then split in nine aliquots (technical replicates) before the incorporation of the corresponding amount of MC (Table 3) and subsequent DNA extraction.

*Lumbricus* spp. individuals were collected by hand sampling ^[3]^. Single animals were grounded to powder with a sterile mortal containing liquid nitrogen and a sterile pestle. Approximately 10mg of frozen animal powder was transferred in Eppendorf™ Safe-Lock Tubes, Forensic DNA Grade (Fisher Scientific) to generate multiple replicated aliquots of the same animal. DNA extractions were performed from three individuals; the powder of each individual was investigated using three technical replicates with a different amount of MC (Table 3).

Alive Carabidae were collected by hand sampling ^[3]^. Following the taxonomic identification using morphological keys ^[4]^, the animals were rinsed with a in 0.1 M Tris Saline Buffer to remove soil and stored at -20°C. For DNA extraction, a single individual was homogenized in 100ml sterile DNA/DNAse free water (Merck, Germany) by using a 5mm Stainless Steel Bead (QIAGEN) on a TissueLyser II (QIAGEN). For each animal (N: 3), the homogenate was then split in four aliquots (replicates) before the incorporation of the corresponding amount of MC and subsequent DNA extraction. DNA extractions were performed from three individuals; each individual was investigated using three technical replicates with a different amount of MC (Table 3).

Collembola were heat-extracted from soil cores by using a modified Kempson apparatus ^[5]^, rinsed with a in 0.1 M Tris Saline Buffer to remove soil and stored at -20°C. For DNA extraction, 6 individuals were homogenized in 100ml sterile DNA/DNAse free water (Merck, Germany) by using a 5mm Stainless Steel Bead (QIAGEN) on a TissueLyser II (QIAGEN). The homogenate was then split in six aliquots (technical replicates) before the incorporation of the corresponding amount of MC and subsequent DNA extraction (Table 3).

Nematoda were extracted from soil by using a Baermann funnel extraction protocol ^[6]^. For DNA extraction, nematodes were pooled in three samples of approximately 90 individuals per sample. Samples were homogenized in 100ml sterile DNA/DNAse free water (Merck, Germany) by using a 5mm Stainless Steel Bead (QIAGEN) on a TissueLyser II (QIAGEN). The homogenate of each pool was then split in three aliquots (replicates) before the incorporation of the corresponding amount of MC and subsequent DNA extraction (Table 3).

DNA extractions from test samples were performed with the kit NucleoSpin® Soil mini kit (MACHEREY-NAGEL) as reported by ^[2]^. Sample and MC co-extraction has been performed by using ZymoBIOMICS™ Spike-in Control I as MC and by using the MC doses detailed in Table 3. Additional information regarding the quantification of each DNA extract is reported on table S4. The amplification of the 16S rRNA gene region V3-V4 was performed as described in (<https://earthmicrobiome.org/protocols-and-standards/16s/>), by using the KAPA HiFi HotStart ReadyMix (Roche Applied Science), with the two primers 341F_ILL and 805R_ILL ^[7],[8]^. Additional details regarding PCR conditions, such as the amount of DNA incorporated in each PCR reaction and the number of PCR cycles, are reported in Table S4. High-throughput sequencing of the amplicon libraries using Illumina technology were performed at the Genomics Platform, Fondazione E. Mach. The 57 amplicon libraries were sequenced Illumina (Illumina, UK) MiSeq Standard Flow Cells using 600 cycle V3 reagents and with a minimum depth of 30,000 reads per sample. Bioinformatic processing of fastq files and statistical analyses were performed as described for bovine fecal pools in the materials and methods.

To independently quantify the number of copies of the 16S rRNA target region (V3-V4), for each replicate, digital-droplet PCR (ddPCR) was performed as previously described ^[9]^, using the primer pair 341f (CCTACGGGNGGCWGCAG) and 805r (GACTACNVGGGTWTCTAATCC) (31) on a QX200TM droplet digital PCR system using a QX200TM droplet generator (Bio-Rad, Munich, Germany). Droplets were analysed in the QX200TM droplet reader using the QuantaSoftTM Analysis Pro software (Bio-Rad, Munich, Germany). Droplet distribution was checked to ensure a clear separation of positive and negative droplets.

The plot displaying the correlation between Log_2_ 16S rRNA gene copies estimated by ddPCR (y axis) and miSeq (x axis) was generated using R package ggplot2 ^[10]^. The linear regression model was generated using the lm function of the R stats package in R ^[11]^.

References

1. Barillot, C. D. C., Sarde, C.-O., Bert, V., Tarnaud, E. & Cochet, N. A standardized method for the sampling of rhizosphere and rhizoplan soil bacteria associated to a herbaceous root system. *Ann. Microbiol. 2012 632* **63**, 471–476 (2013).

2. Praeg, N., Pauli, H. & Illmer, P. Microbial Diversity in Bulk and Rhizosphere Soil of Ranunculus glacialis Along a High-Alpine Altitudinal Gradient. *Front. Microbiol.* **0**, 1429 (2019).

3. Moret, P., Aráuz, M. de los Á., Gobbi, M. & Barragán, Á. Climate warming effects in the tropical Andes: first evidence for upslope shifts of Carabidae (Coleoptera) in Ecuador. *Insect Conserv. Divers.* **9**, 342–350 (2016).

4. Pesarini, C. Monzini, V. Insetti della fauna italiana. Coleotteri Carabidi I. Natura. https://scholar.google.com/scholar?hl=it&as_sdt=0%2C5&q=Insetti+della+Fauna+italiana.+Coleotteri+Carabidi+I.+Natura.+&btnG=.

5. KEMPSON, Denys, LLOYD, M. & GHELARDI, R. A new extractor for woodland litter. *Pedobiologia (Jena).* **3**, 1–21 (1963).

6. Viglierchio, D. R. & Schmitt, R. V. On the Methodology of Nematode Extraction from Field Samples: BaermannFunnel Modifications. *J. Nematol.* **15**, 438 (1983).

7. Klindworth, A., *et al.* Evaluation of general 16S ribosomal RNA gene PCR primers for classical and next-generation sequencing-based diversity studies. *Nucleic Acids Res.* **41**, e1–e1 (2013).

8. Apprill, A., McNally, S., Parsons, R. & Weber, L. Minor revision to V4 region SSU rRNA 806R gene primer greatly increases detection of SAR11 bacterioplankton. *Aquat. Microb. Ecol.* **75**, 129–137 (2015).

9. Praeg, N., Schwinghammer, L. & Illmer, P. Larix decidua and additional light affect the methane balance of forest soil and the abundance of methanogenic and methanotrophic microorganisms. *FEMS Microbiol. Lett.* **366**, 259 (2019).

10. Wickham, H. ggplot2 Elegant Graphics for Data Analysis Second Edition. (2016).

11. Team, R. C. R: A Language and Environment for Statistical Computing. (2019).
