# Supplementary material for: Mock community as an in situ positive control for amplicon sequencing of microbiotas from the same ecosystem
Source: Sci Rep. 2023 Mar 11;13:4056. doi: 10.1038/s41598-023-30916-1 (PMC10008532; doi:10.1038/s41598-023-30916-1)
Supplement: Supplementary file 2 — Supplementary Information 2. [file 41598_2023_30916_MOESM2_ESM.pdf]

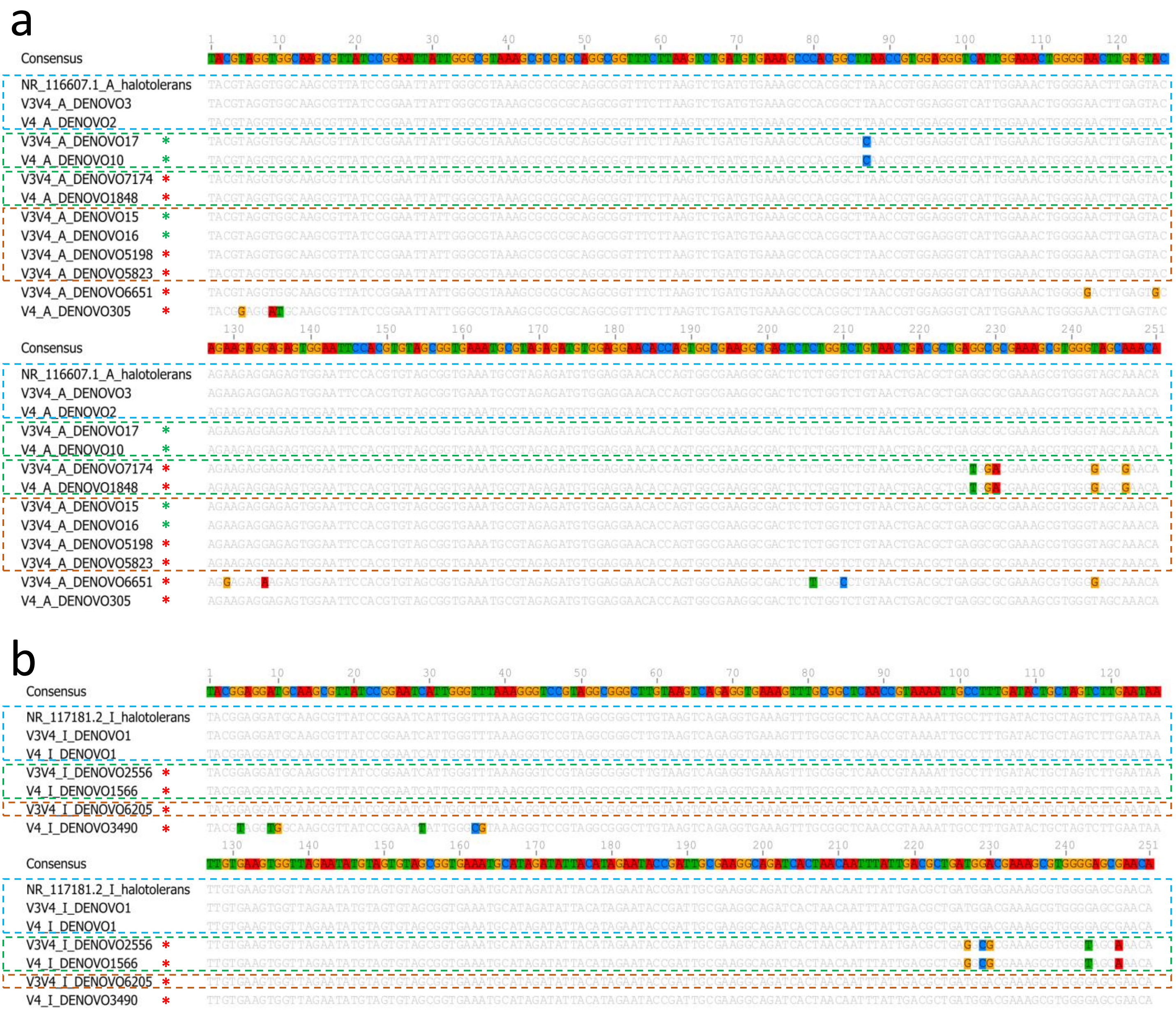

**Figure S1**. Multiple sequence alignments of *A. halotolerans* (a) and *I. halotolerans* (b) MC-SVs. SVs were amplified using V4 (515_ILL – 806_ILL) and V3-V4 (341_ILL – 805_ILL) primer pairs in independent experiments (MC co-extracted with bovine pools and test samples, respectively). MC-related SVs were aligned to the publicly available sequences (*A. halotolerans*: NR_116607.1; *I. halotolerans*: NR_117181.2) and trimmed to the overlapping V4 region. SVs were named with the corresponding primer pair (V4; V3-V4), followed by an MC taxa identifier (A: *A. halotolerans*; I: *I. halotolerans*) and the original SV name (e.g. DENOVO1). Blue boxes indicate primary SVs (e.g. MC-SVs with the highest abundance and sharing 100% identity with the corresponding reference sequence). Green boxes indicate SVs amplified with both primer pairs (proportion of reads compared to primary SVs ranging from 0.3 to <0.001). Orange boxes indicate V3-V4 sequence variants sharing 100% identity with the corresponding V4 reference sequence but polymorphic in the V3 region (proportion of reads compared to primary ASVs ranging from 0.3 to <0.001). No box indicates MC-related SVs detected with only one primer combination and sharing <100% identity with the corresponding reference in the V4 region (proportion of reads compared to primary ASVs ranging from 0.01 and <0.001). *: proportion of reads compared to primary ASVs < 0.01. * : proportion of reads compared to primary ASVs > 0.2. The proportion of reads of reads was estimated as the ratio between the abundances of MC-SVs

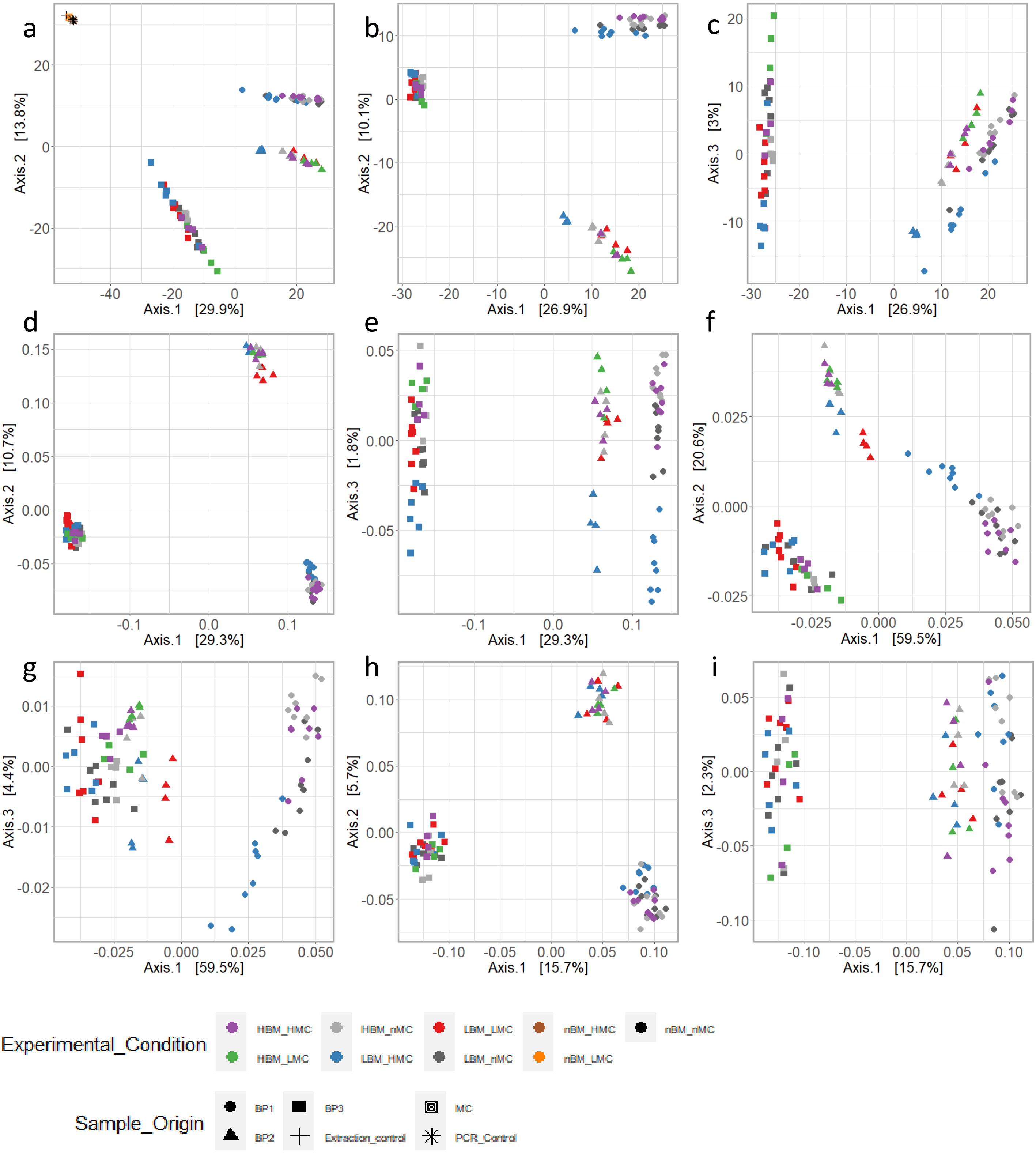


**Figure S2**. Beta diversity estimates across bovine faecal pools (BP1, 2, 3) with high and low biomasses and MC dose. **a**: extraction Controls (lysis buffer only: no fecal material and no MC), PCR controls (PCR buffer only: no DNA template) and MC controls (MC only: no fecal sample) were also included in the PCoA. PCoAs were generated with the following distance metrics: **a**, **b**, **c**: Aitchison distance; **d**, **e**: Bray-Curtis dissimilarity; **f**, **g**: weighted UniFrac; **h**, **i**: unweighted UniFrac. For each distance metrix, plots **a**, **b**, **d**, **f**, **h** display the ordination according to Component 1 and Component 2, while plots: **c**, **e**, **g**, **i** display the ordination according to Component 1 and Component 3.

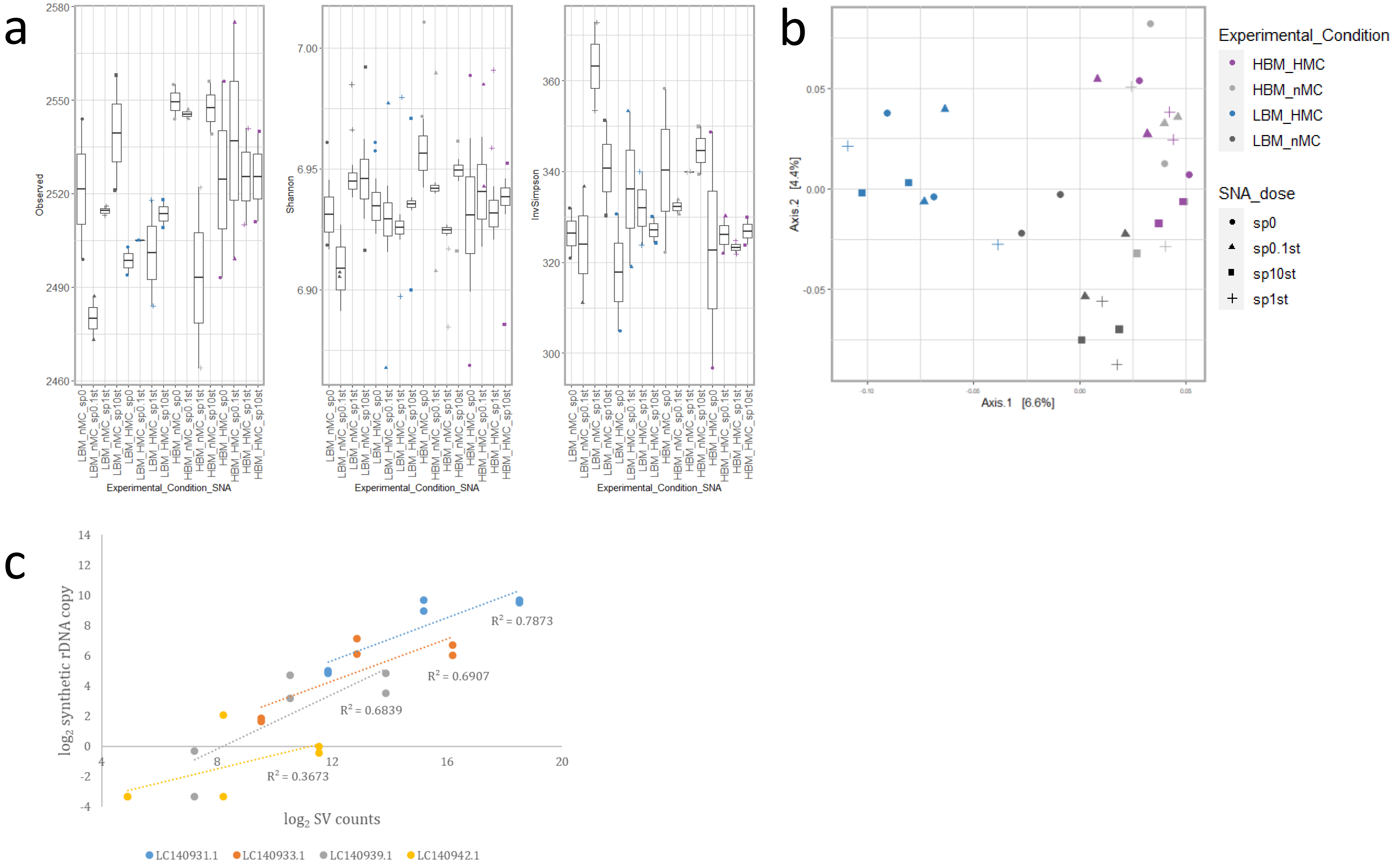


**Figure S3**. Diversity estimates across BP1 faecal pools with high and low biomasses, and high and low doses of MC and synthetic rDNA molecules. **a**: alpha diversity estimates; **b**: Bray-Curtis dissimilarity estimates for BP1 replicates. **c**: Correlation between the theoretical number of SNA copies in amplification reactions (y axis) and the observed number of synthetic rDNA-derived SVs. SV counts were log2(x+0.1) transformed.

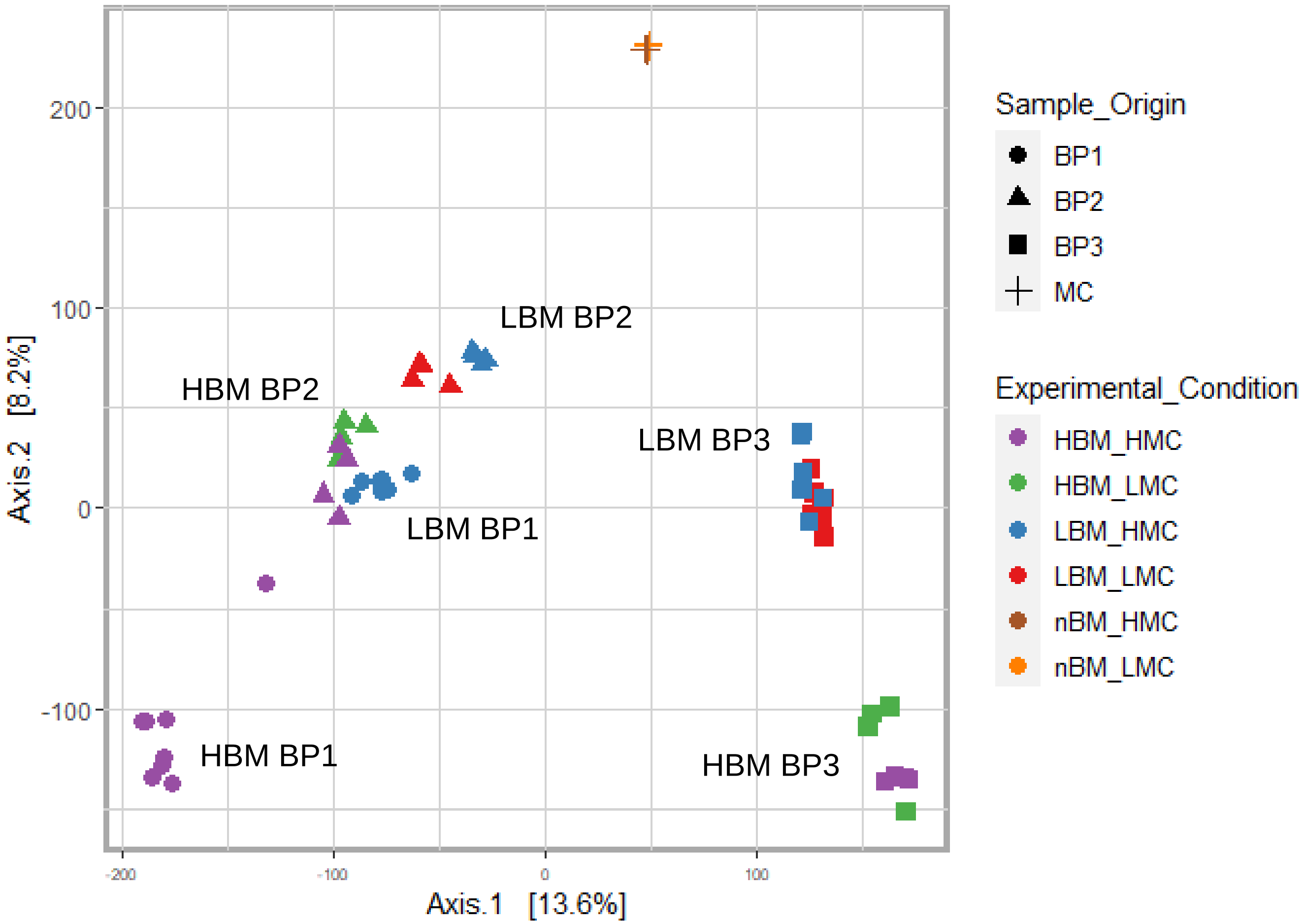


**Figure S4**. Beta diversity estimates in bovine faecal samples (pools BP1, 2, 3). SVs counts were transformed according to the MC-SV abundance.Sample-SVs counts were transformed according to the abundance of MC-SVs. PCA were generated by using Euclidean distance. SV counts were transformed according to the MC-SVs in each library

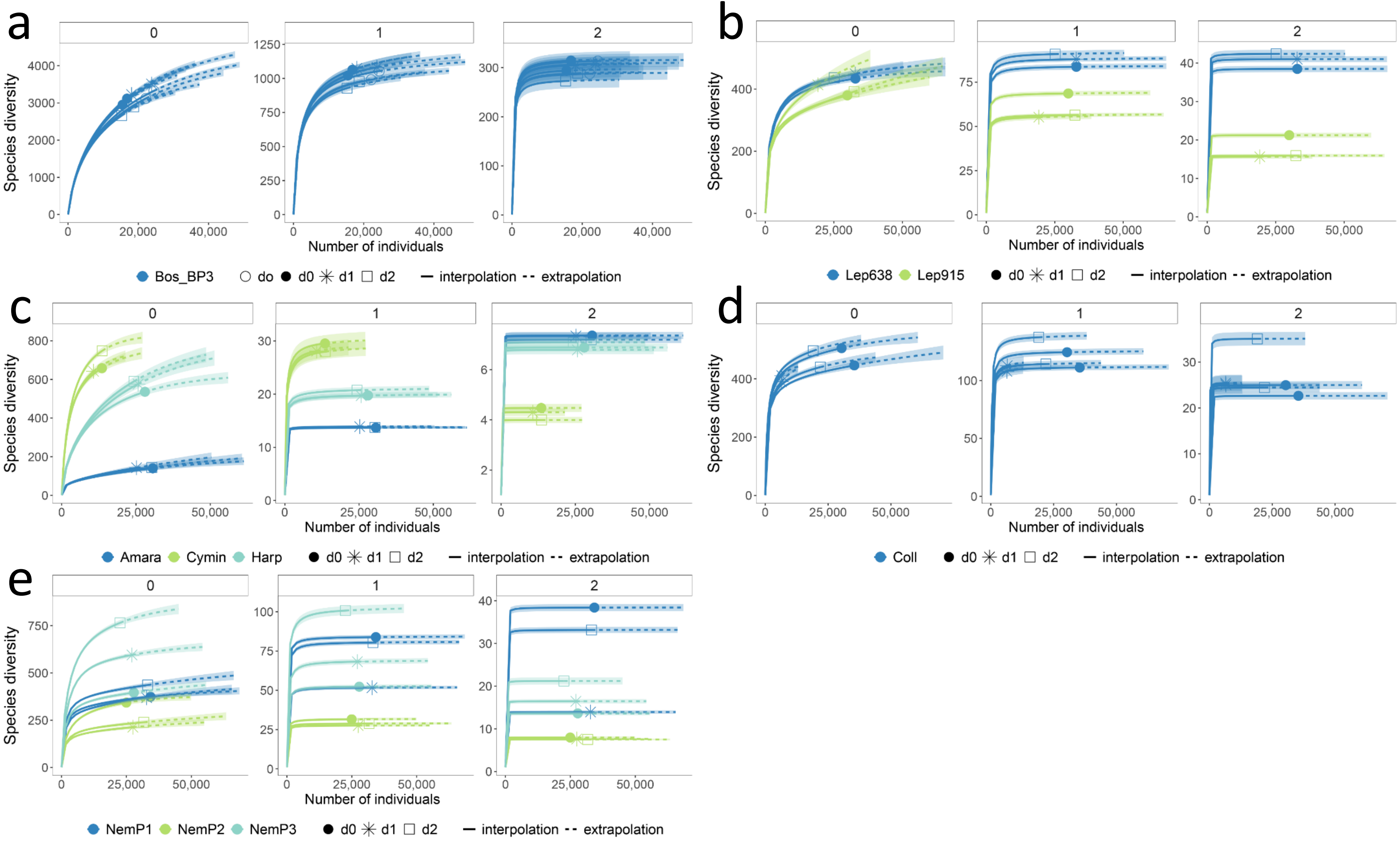


**Figure S5**. Sample-size-based rarefaction (solid lines) and extrapolation (dotted lines) sampling curves for test samples. For each sample type, rarefaction and extrapolation curves were separated by the diversity order [q]: q = 0 (species richness, left panel), q = 1 (Shannon diversity, middle panel) and q = 2 (Simpson diversity, right panel). 95% confidence intervals based on a bootstrap method with 200 replications are shown as shaded areas. **a**: *B. taurus*, **b**: *L. europeaus*, **c**: Coleoptera, **d**: Collembola, **e**: Nematoda

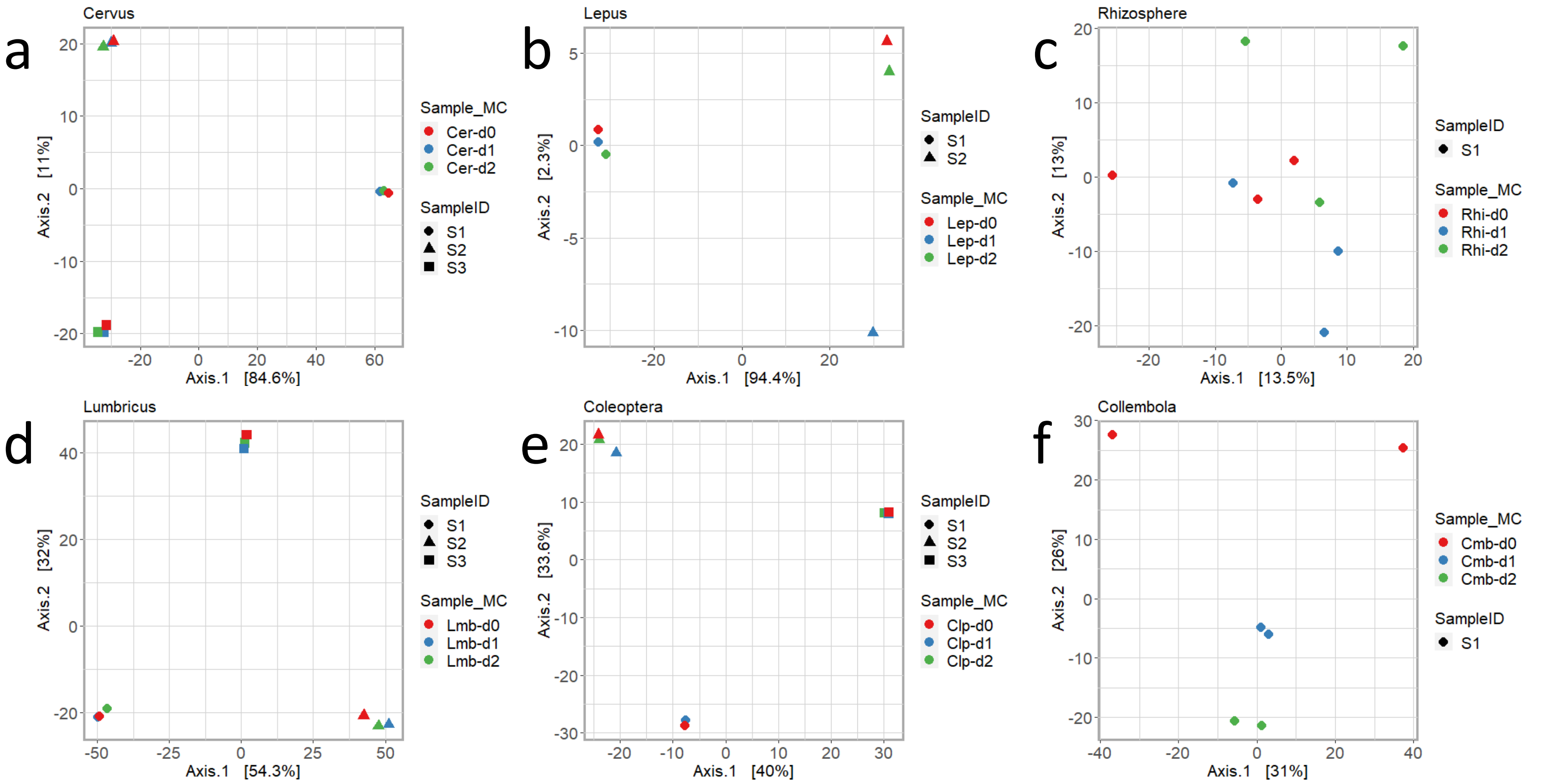

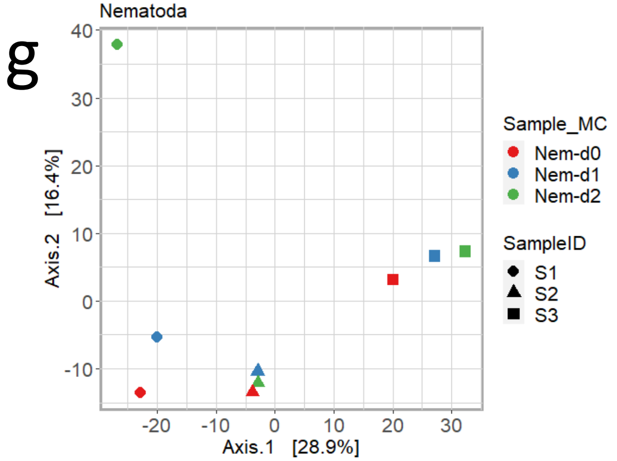


**Figure S6**. Beta diversity estimates across test samples with high and low MC doses. **a**: *C. elaphus*, **b**: *L. europaeus*, **c**: *Carex* spp. rhizosphere soil, **d**: *Lumbricus* spp., **e**: Coleoptera, **f**: Collembola, **g**: Nematoda.
